# Garmin GPSMAP 66sr: Assessment of Its GNSS Observations and Centimeter-Accurate Positioning

**DOI:** 10.3390/s22051964

**Published:** 2022-03-02

**Authors:** Lambert Wanninger, Anja Heßelbarth, Volker Frevert

**Affiliations:** Geodätisches Institut, School of Civil and Environmental Engineering, Technische Universität Dresden, D-01062 Dresden, Germany; anja_brit.hesselbarth@tu-dresden.de (A.H.); volker.frevert@tu-dresden.de (V.F.)

**Keywords:** Garmin GPSMAP 66sr, dual-frequency GNSS, carrier-phase observations, ambiguity fixing, phase-center calibration, postprocessing, centimeter-accurate positioning, precise point positioning (PPP), virtual reference station (VRS)

## Abstract

In 2020, Garmin released one of the first consumer devices with a dual-frequency GNSS chip and a quadrifilar helix antenna: GPSMAP 66sr. The device is intended to serve as a positioning and navigation device for outdoor recreation purposes with positioning accuracies on the few meter level. However, due to its highly accurate GNSS dual-frequency carrier-phase observations, the equipment can also be used for centimeter-accurate positioning. We performed extensive test measurements and analyzed the quality of its code and carrier-phase observations. We calibrated the Garmin GPSMAP 66sr antenna with respect to its phase-center offset and phase-center variations. We also performed dual-frequency GPS/Galileo precise point positioning (PPP) and precise relative positioning in baselines to virtual reference stations (VRS). We demonstrate and explain how centimeter-accurate positioning can be achieved with this novel kind of equipment.

## 1. Introduction

In recent years, an increasing number of consumer devices have been released that possess dual-frequency GNSS chips and make code and carrier-phase observations available. Most of these devices are smartphones, and for some of these smartphones, it could be shown that GNSS carrier-phase ambiguity fixing is feasible and, thus, centimeter-accurate positions can be obtained, at least under favorable conditions [1,2,3]. The main drawback of smartphones with respect to GNSS originates from their simple nonpolarized antennas, which are unable to mitigate multipath signals.

**Figure 1:**
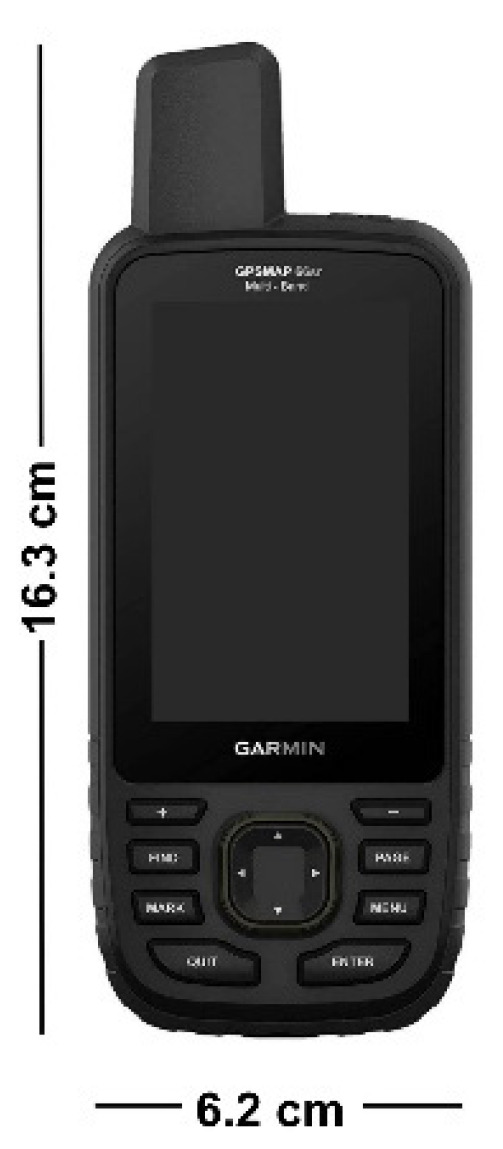
Garmin GPSMAP 66sr with its quadrifilar helix antenna at the top left.

In August 2020, Garmin introduced a new GNSS positioning and navigation device to their product line of outdoor equipment for recreational purposes: Garmin GPSMAP 66sr (Figure 1). It features a multi-GNSS chip, capable to observe GPS, GLONASS, Galileo, and QZSS satellite signals and it even performs dual-frequency observations on GPS, Galileo, and QZSS. It is advertised as a handheld multi-frequency GNSS device that enables improved user position accuracy, specifically in areas where GNSS signals are reflected weak or typically do not penetrate [4]. This statement refers to the meter-accurate stand-alone standard positioning technique. The feature we were interested in, however, is the ability to provide not just code observations but also carrier-phase observations, thus the chance to produce even centimeter-accurate positioning results.

A great advantage of the GPSMAP 66sr in comparison with other consumer devices with dual-frequency GNSS chips, e.g., smartphones such as Xiaomi Mi8 or Huawei P30 [5], consists of its quadrifilar helix antenna (QHA). QHA produces a circularly polarized hemispherical radiation pattern and, thus, is able to mitigate multipath signals [6].

It proved to be a major advantage of the equipment that GNSS observations can be stored internally in RINEX 3.04 format [7] and that these files can be easily transferred to a computer for postprocessing. The GPSMAP 66sr is not designed to allow centimeter-accurate GNSS real-time positioning on the device itself. Therefore, it should be considered as a GNSS receiver with the potential to obtain centimeter-accurate positions in postprocessing only.

In Section 2, we provide an overview of the observation data we gathered, as well as the data processing. Section 3 deals with the assessment of the observation quality and antenna phase-center calibration. In Section 4, we demonstrate how centimeter-accurate positions can be obtained with GPSMAP 66sr either with precise point positioning (PPP) or in baseline mode with respect to a virtual reference station (VRS).

Throughout this paper, we identify the GNSS satellite signals by two-character codes: a letter that describes the satellite system (G—GPS, R—GLONASS, E—Galileo) and a digit for the frequency number (1, 2, or 5), following the RINEX format.

## 2. Observation Data and Data Processing

We performed and recorded several 100 h of GNSS observations with GPSMAP 66sr. Most of these datasets were collected in a roof-top environment without any signal obstructions and with low multipath levels (Section 3.1, Section 3.2, Section 3.3 and Section 4.1). Further datasets originate from field measurements under adverse environmental conditions (Section 4.1 and Section 4.2). All these observations were performed in static mode, mostly at sites with precisely known coordinates from earlier determinations with geodetic-grade GNSS equipment.

All GPSMAP 66sr observations analyzed and discussed in this paper were performed, with the latest firmware available when we bought the receiver in June 2021: Garmin software v3.30/GPS software v2.00.05.

The observation data were recorded internally in RINEX 3.04 and transferred to a computer using the micro-USB (universal serial bus) connector. The data interval was fixed to 1 s. For many processing steps, we reduced the observation interval to 15 s after the data transfer. RINEX navigation files are not recorded by GPSMAP 66sr. Thus, we had to obtain appropriate RINEX navigation files from other sources, e.g., from NASA’s archive of space geodesy data CDDIS [8].

The internal, rechargeable built-in lithium-ion battery lasted for at least 16 h in full GNSS mode (dual-frequency, all systems). For longer observation sessions, we used the external power supply via micro-USB.

Centimeter-accurate positioning in precise point positioning (PPP) mode or in relative mode requires additional data, such as precise GNSS orbit and clock information or GNSS observations from reference stations. For analysis steps that required a local GNSS reference station, we used a Septentrio PolaRx5 receiver connected to a choke ring antenna of type Javad RingAnt-DM JVDM. Additional datasets are described, and their sources are listed in the following Section 3 and Section 4.

All analysis steps presented in Section 3 were performed with software modules belonging to the WaSoft software package [9]. Our original intention was to produce positioning results using free processing services. However, we failed to obtain such results in the case of PPP. Therefore, we used the WaSoft module WaPPP instead. Centimeter-accurate baseline results with respect to a VRS could be obtained from the free processing service SAPOS GPPS-PrO. Further details are provided in Section 4.

## 3. Assessment of Observation Quality and Antenna Calibration

In preparation of centimeter-accurate positioning, we analyzed the observation data with respect to the completeness, data quality of dual-frequency code and carrier-phase observations (Section 3.1), and the integer property of the carrier-phase observation in baselines to geodetic-grade equipment (Section 3.2). Since ambiguity fixing could be performed successfully, we were able to calibrate the antennas with respect to their mean phase center and with respect to phase-center variations (Section 3.3).

### 3.1. Observation Quality

**Figure 2:**
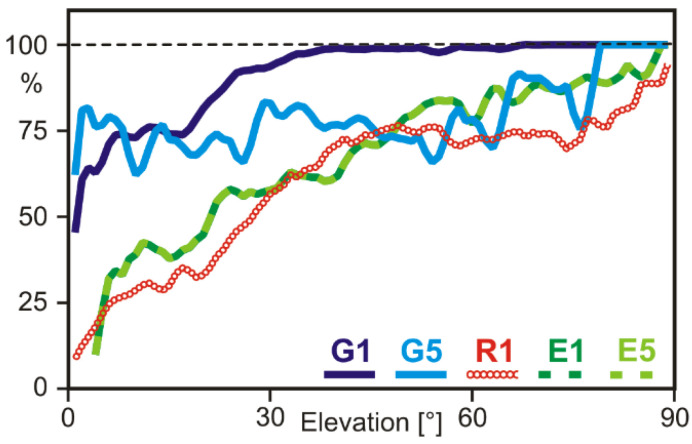
Completeness of GPSMAP 66sr GNSS observations as function of elevation angle and GNSS signal.

GNSS observations of the Garmin GPSMAP 66sr (software v3.30) consist of code, carrier phase, Doppler, and C/N0 on one (GLONASS) or two frequencies (GPS, Galileo, QZSS). However, the datasets are not complete, but the tracking channels seem to be assigned to certain kinds of satellites and frequencies with different priorities. Based on long-term static observations at a roof-top site without any signal obstructions, we determined the completeness of the observations as percentages of the available GNSS signals (Figure 2). GNSS satellites that were set to an unhealthy status were excluded. QZSS satellites were also discarded since the sample collected at the central European site was too small to obtain meaningful results.

**Figure 3:**
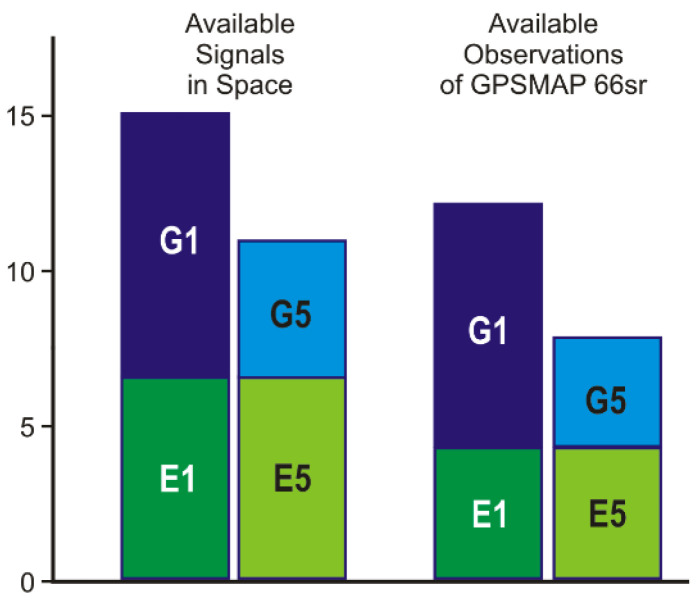
Average number of available GPS/Galileo signals and actual GPSMAP 66sr observations per epoch at a mid-latitude site (Dresden) without any signal obstructions in mid-2021, elevation mask angle 10 deg.

The highest percentage of complete observation datasets was obtained for the first frequency of GPS. Above an elevation mask angle of 30 degrees, the observations are virtually complete. However, around 25% of the satellites elevated between 10 and 20 degrees were not observed. The missing number of observations of the second GPS frequency G5 reaches around 25% for almost all elevation angles. Here, it has to be taken into account that in 2021 only about half of the satellites in the present GPS constellation are able to transmit G5 signals. Thus, the overall number of dual-frequency GPS observations amounts to less than 50% of the available G1 observations (Figure 3).

The number of Galileo observations on both frequencies is identical (Figure 2 and Figure 3), but their completeness is very much elevation-dependent. On average, only about 60% of the available signals were observed. The maximum number of simultaneously observed Galileo signals never exceeded seven satellites, even if more satellites were far above the horizon. The settings of the GNSS chip seem to restrict the number of Galileo observations, although they could contribute very much to high-accurate dual-frequency observations.

GLONASS single-frequency observations are also incomplete, with only about 50% of the available signals being observed. Due to frequent interruptions of the signals’ tracking, which results in observation gaps and cycle slips, GLONASS observations could only be considered in some of the quality further assessment steps and were not taken into account in Figure 3.

Figure 3 compares the average number of available signals in space with the number of actually observed signals. Presently (mid-2021), around 15 GPS/Galileo signals are available on G1/E1 and 11 signals on G5/E5. The GPSMAP 66sr (v3.30), however, observes on average only 12 satellites on the first frequency and 8 satellites on the second. Due to the continuously changing satellite distribution, the number of dual-frequency observations can occasionally drop below five, even at sites without any signal obstructions. Under less favorable conditions, the number of observed satellites often falls short of the minimum number of required satellites. This occurs more often for G5/E5 or dual-frequency observations, compared with G1/E1 single-frequency observations.

**Figure 4:**
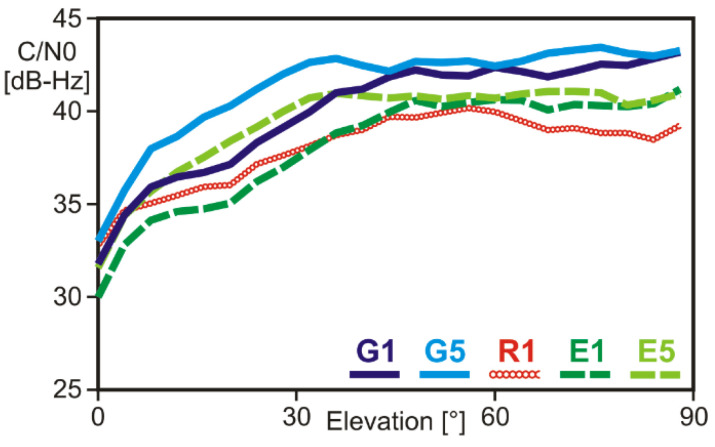
Carrier-to-noise power density ration C/N_0_ of the available GPSMAP 66sr observations as a function of elevation angle and GNSS signal.

The recorded signal strength values C/N_0_ show the typical elevation dependence known from the patch and other helix antennas (Figure 4). Maximum signal levels are reached for elevation angles larger than 40 deg. Below elevation angles of 20 deg, the signal strength decreases rapidly with decreasing elevation angle. On average, GPS signal strength levels exceed those of Galileo and GLONASS signals. G5/E5 signal levels are higher than the corresponding G1/E1 signals.

**Figure 5:**
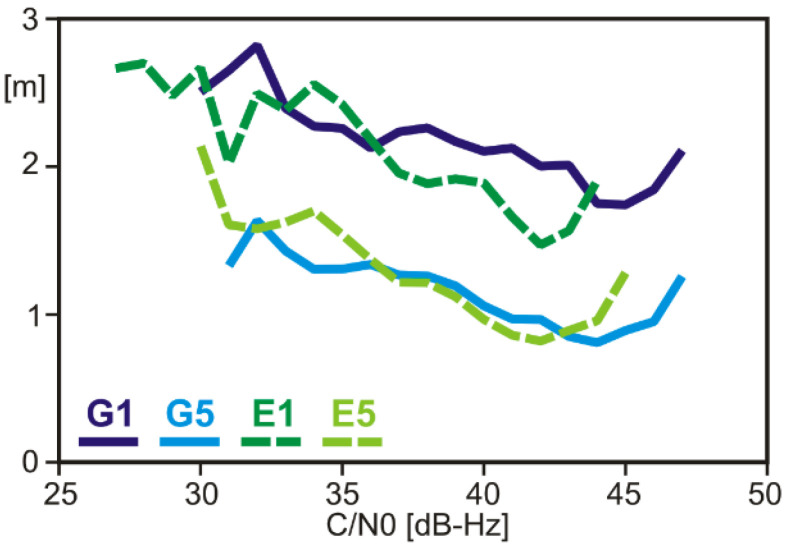
Code noise and multipath of the GPSMAP 66sr, determined from code-minus-carrier linear combination of dual-frequency signals (RMS values).

Code noise and multipath levels were estimated using the MP (multipath) linear combination of code observations and dual-frequency carrier-phase observations, which is sometimes also called code-minus-carrier (CMC) linear combination [10]. These observations were collected in a roof-top environment with little multipath disturbances. The results reveal a considerable difference between G1/E1 and G5/E5 (Figure 5). The code noise and multipath levels of the G5/E5 signals are just half as large as those of G1/E1, which can be attributed to the improved signal structure of G5/E5, compared with G1/E1. Only small differences exist between GPS and Galileo.

**Figure 6:**
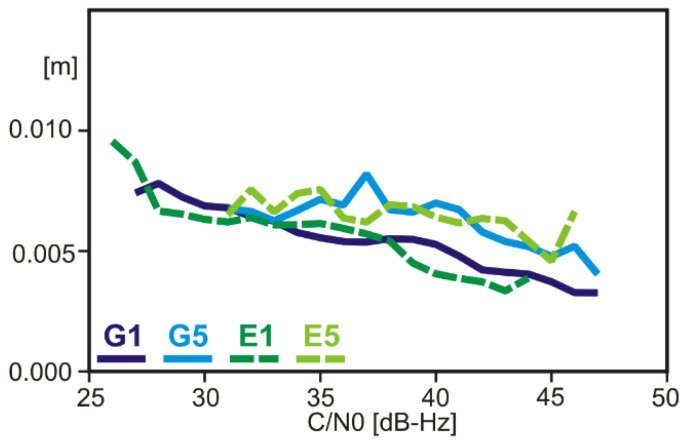
Carrier-phase noise and multipath of the GPSMAP 66sr as determined from the observation residuals of a short baseline of two such receivers (RMS values).

Carrier-phase noise and multipath levels were estimated using a short (few meters long) baseline of two Garmin GPSMAP 66sr deployed in a roof-top environment with little multipath disturbances. Differences among the four signals are fairly small (Figure 6), with slightly higher RMS values for G5/E5 than those for G1/E1. This difference can be explained by the slightly longer wavelength at the G5/E5 frequency.

Although the GNSS observations collected by GPSMAP 66sr are by far not complete, they are of high quality for both main observables: code and carrier phase. Code measurements on G5/E5 reveal a noise and multipath level at a favorable site of around 1 m, only half as high as for G1/E1. Carrier-phase observations reveal noise and multipath variations in the order of several millimeters.

### 3.2. Integer Property of Estimated Ambiguities

Double-difference carrier-phase ambiguities must possess integer property to make the very high accuracy of the carrier-phase observations available to positioning without any degradation and, thus, to centimeter-accurate positioning results. In contrast to geodetic-grade receivers, which always produce carrier-phase observations with the property of integer ambiguities, other equipment, e.g., smartphones, can fail in this respect [2].

Double-difference (DD) carrier-phase observations involve simultaneous GNSS observations of two receivers. In order to test the GPSMAP 66sr with respect to the integer property of its carrier-phase ambiguities, we processed a 24 h dataset of a short, known baseline (7 m) between a geodetic-grade Septentrio PolaRx5 and a GPSMAP 66sr. The data were collected in a roof-top environment with little multipath disturbances. Antenna phase-center corrections were applied (Section 3.3), and the observations were processed in baseline mode.

Further analysis was conducted based on DD residuals of the carrier-phase observations. Due to the very short length of the baseline, they are free of atmospheric effects and not affected by orbit errors. Thus, the information content of the DD residuals comprises carrier-phase multipath, noise, and eventually, differential instrumental delays, which may prevent integer ambiguities.

**Figure 7:**
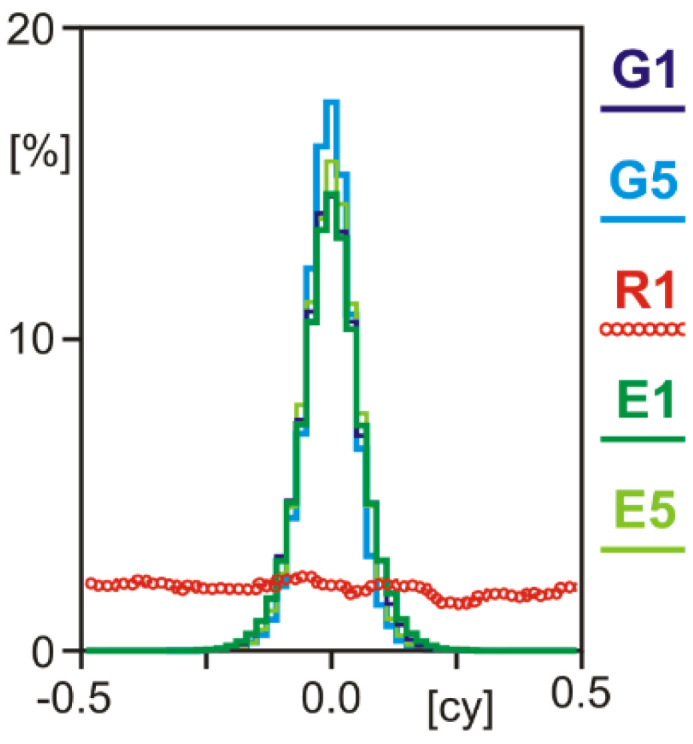
Distribution of single-epoch double-difference fractional cycle ambiguities of a short, known baseline between geodetic-grade equipment and GPSMAP 66sr.

The property of integer ambiguities can be detected best by a statistical analysis of fractional cycle DD residuals. In the case of integer property, their distribution shows a clear maximum at 0.0 cycles. Distributions without a clear maximum indicate that no integer ambiguity fixing can be performed with this signal. In the case of the GPSMAP 66sr, the GPS and Galileo carrier-phase observations clearly prove their integer ambiguity property, whereas the GLONASS R1 signal does not, which is probably caused by the FDMA technique used for GLONASS signals (Figure 7). Similar results had been found for selected smartphones in recent years [5], but compared with the smartphones, the GPSMAP 66sr produces smaller deviations of the GPS/Galileo residuals from zero, which is suspected to be caused by its higher-quality antenna.

**Figure 8:**
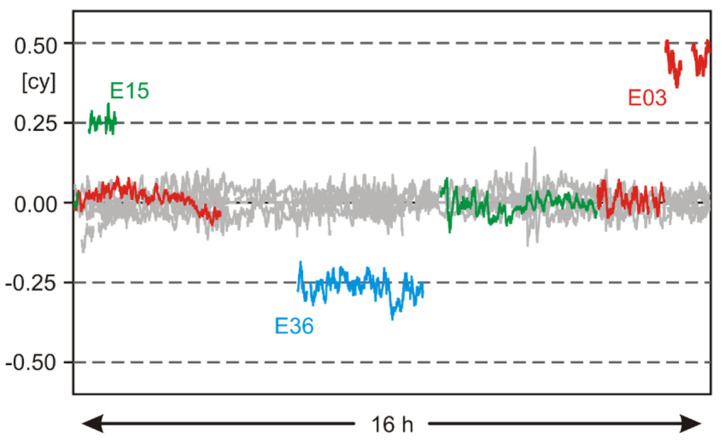
Single-difference G5/E5 residuals of a short, known baseline between geodetic-grade equipment and GPSMAP 66sr. Epoch-wise differential receiver clock errors were estimated from ambiguity-fixed observations and removed for all the observations. Residuals of those three satellites with temporary quarter- or half-cycle ambiguities are shown in colors. All other GPS/Galileo residuals are shown in gray.

When processing several long sessions of GPSMAP 66sr carrier-phase observations, we found that some G5/E5 ambiguities were not integers but seem to be a quarter- or half-cycle off the full integer (Figure 8). In the RINEX observation files, these observations had not been flagged accordingly.

When processing a static baseline, this results in an ambiguity fixing of all carrier-phase observations except those with quarter- or half-cycle offsets. As long as only very few observations are affected by this problem, static positioning is not degraded. However, in precise kinematic baseline processing, such errors cause incorrect ambiguity fixing and, consequently, incorrect positioning results. Due to these incorrect carrier-phase observations, we were not able to produce correct and reliable centimeter-accurate kinematic results.

### 3.3. Antenna Phase-Center Calibration

**Figure 9:**
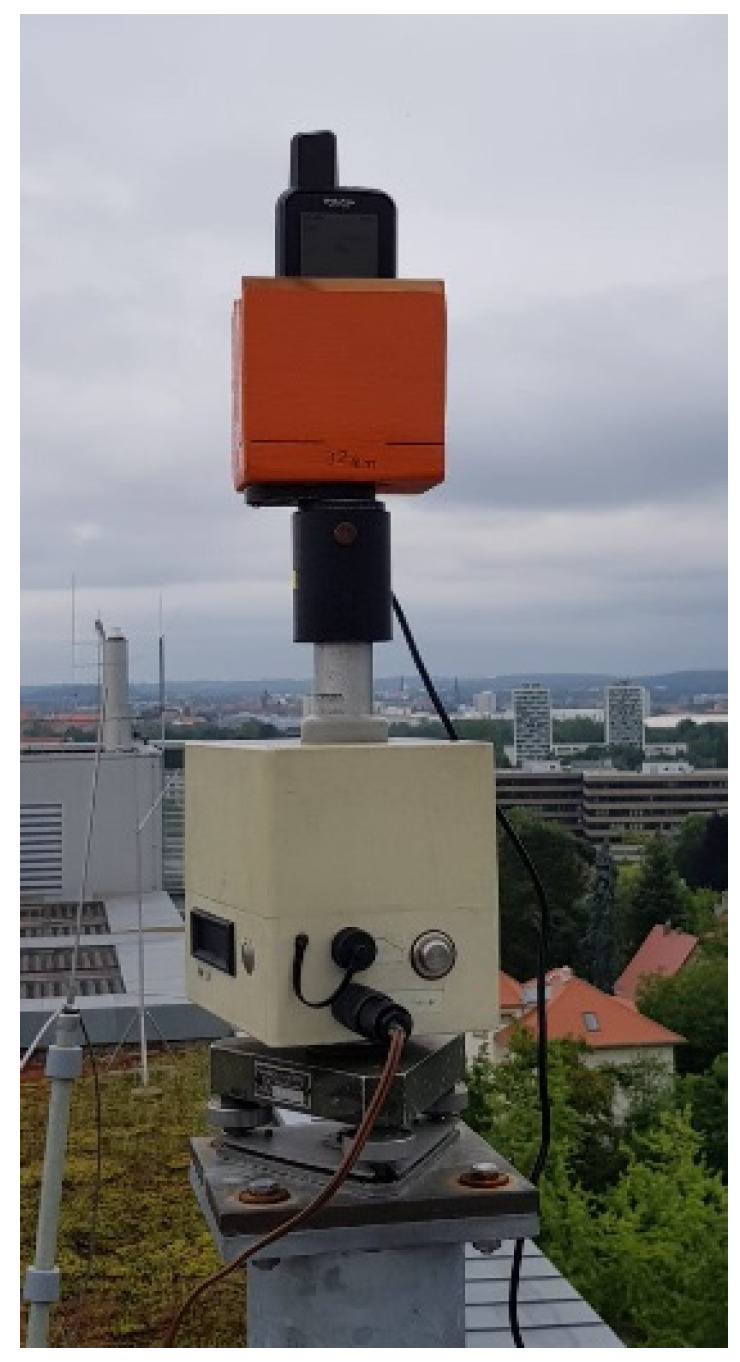
GPSMAP 66sr in its holder on top of the DRB2 rotational device, which enables observations in four azimuthal orientations per minute.

**Figure 10:**
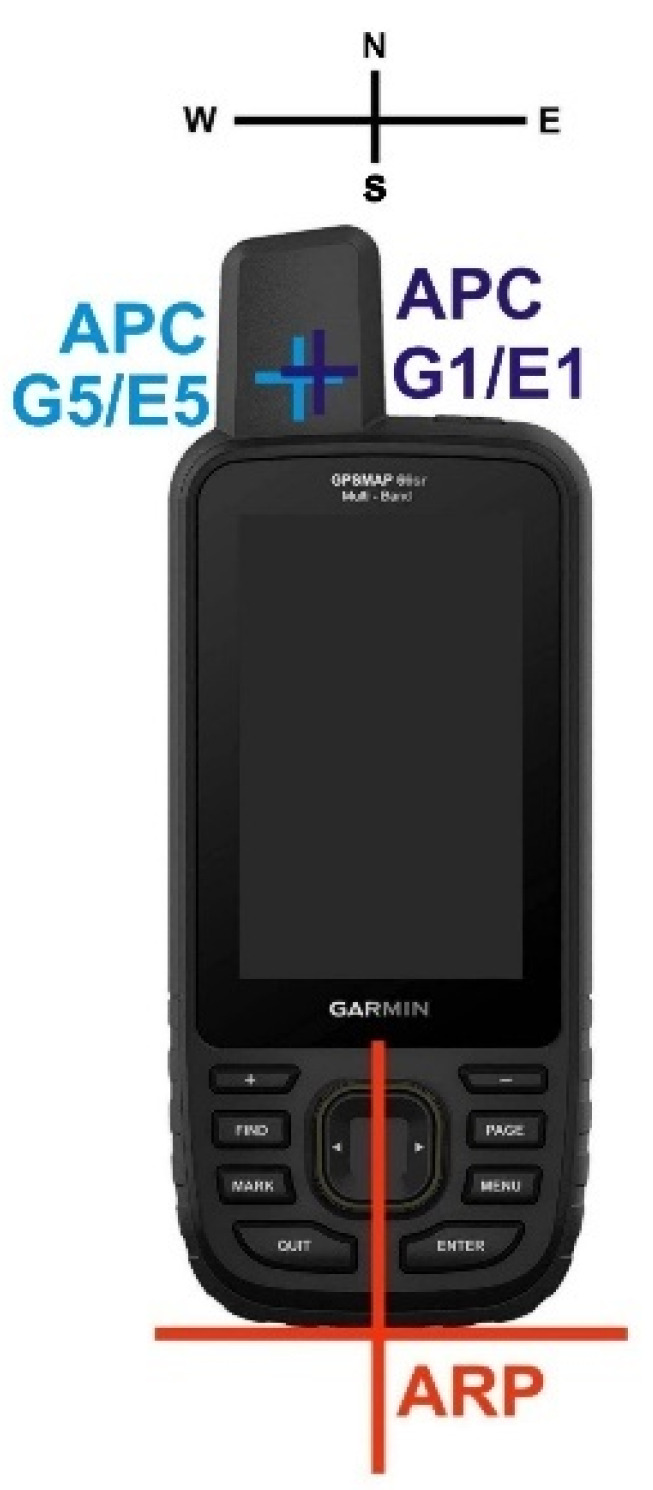
Depiction of the necessary specifications with regard to antenna phase center (APC) calibration: position of the antenna reference point (ARP) and azimuthal orientation of the device toward north. The mean APCs as determined by calibration are also shown.

Phase-center offsets (PCOs) and variations (PCVs) were determined for the GPSMAP 66sr by field calibration. We used the DRB2 rotational device (Figure 9), which enables observations in four azimuthal orientations per minute but does not perform antenna tiltings [11]. The GPSMAP 66sr was mounted in a holder in an upright position. We selected an antenna reference point (ARP) to which the PCO refer and defined a north orientation: the device was oriented toward the north when the display pointed south (Figure 10).

The observations in the various azimuthal orientations enable the determination of PCV for the complete upper hemisphere, and they also help to mitigate carrier-phase multipath effects on the horizontal PCO components. However, the height components may still be affected by local multipath.

The local reference station equipped with Septentrio PolaRx5 receiver and JavRingAnt_DM JVDM antenna served as reference. The height difference between the reference antenna and Garmin was determined by precise leveling. The calibration requires ambiguity fixing in the short baseline between reference antenna and antenna to be calibrated and, thus, were limited to the dual-frequency signals of GPS/Galileo. Since the baseline length between the reference antenna and Garmin amounted to just a few meters, all atmospheric influences were eliminated and, therefore, did not degrade the calibration results. The calibration session lasted 16 h.

**Figure 11:**
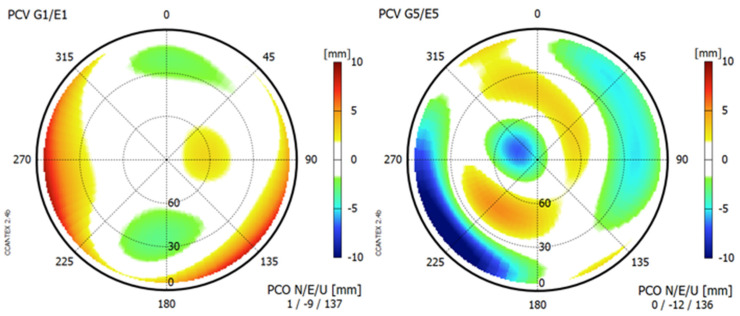
Results of the antenna phase-center calibration of GPSMAP 66sr for G1/E1 (**left panel**) and G5/E5 (**right panel**).

Figure 11 lists PCO and depicts PCV results for GPS/Galileo. The major components to be taken into account for precise positioning are the vertical offsets of almost 14 cm above ARP and the eccentric position of the Garmin antenna with around 1 cm offset to the west. They are also shown in Figure 10. All other calibration components are fairly small: the north offset does not exceed 1 mm, and all PCVs stay below 1 cm. For most precise applications, it will be sufficient to apply corrections for the PCO components only. The full calibration results are available in ANTEX format [12] as Supplementary Material to this paper.

## 4. Precise Positioning Results

Carrier-phase positioning was performed in two different ways. We present results from the absolute positioning technique PPP (Section 4.1) and also from relative baseline positioning with respect to a VRS (Section 4.2).

### 4.1. Dual-Frequency GPS/Galileo Precise Point Positioning

Precise point positioning (PPP) is a GNSS positioning technique that uses the carrier-phase observations as primary observables and is able to produce cm level coordinates if dual-frequency observations are available and if precise satellite orbits and satellite clock corrections are introduced into the data processing. It requires long observation times of continuous carrier-phase tracking in order to achieve the highest accuracy level. Often, the carrier-phase ambiguities are treated as real numbers (float solution). With some additional effort, especially consisting of applying corrections for fractional cycle biases, ambiguity fixing to their true integer values may be achieved. This would improve positioning accuracy, especially in the east component. Coordinates results are obtained in the geodetic reference system of the satellite orbits, which presently is the ITRF 2014. Thus, the coordinates are time-dependent and must be provided with a time stamp.

The requirement of dual-frequency observations reduces the number of satellite signals being available with a GPSMAP 66sr, as all pure single-frequency observations (of around half of the GPS constellation and of GLONASS) are discarded. At an ideal mid-latitude observation site, eight dual-frequency measurements are available on average (cp. Figure 3). If signal obstructions exist in the vicinity of the receiving equipment, the number of measurements often falls below the required minimum of signals.

Our intention was to produce PPP results with one of the free processing services which accept RINEX observation files and return ITRF coordinates and statistical quality measures. We tried to process GPSMAP 66sr RINEX files with the following sources:

- CSRS-PPP of National Resources Canada [13];
- Trimble CenterPoint RTX Post-Processing Service [14];
- GNSS Analysis and Positioning Software (GAPS), University of New Brunswick, Canada [15].

However, we failed for various reasons. Often, we received no result at all but only an error message, or the result was based on GPS single-frequency observations only with poor positioning accuracy. The following main reasons were found:

- The processing service does not accept G5 and/or Galileo observations but only the GPS (+GLONASS) traditional signal frequencies 1 and 2;
- The GPSMAP 66sr antenna does not belong to the group of antennas supported by the processing service.

Since the Garmin GNSS observations are presently not processable by the free PPP processing services, we used our own processing module WaPPP to obtain results. Antenna phase-center corrections, as determined in Section 3.3, were applied. We utilized precise satellite orbits and clock corrections from the Center for Orbit Determination in Europe (CODE). Their clock corrections, as all clock corrections from analysis centers of the International GNSS Service (IGS), refer to the ionosphere-free linear combinations G1/G2-P(Y) and E1/E5 [16]. Thus, additional corrections are needed for GPS satellite clock corrections to compensate for differences in signal delays between ionosphere-free linear combinations G1/G2 and G1/G5. These corrections consist of two portions:

- Code biases between different signals (frequencies and modulations), which are determined from the observation data of globally distributed GNSS reference station networks and are available either as differential code biases (DCBs) [17] or as pseudo-absolute code biases (observable-specific bias (OSB)) [18]. We applied the appropriate GPS DCBs produced by DLR and obtained from CDDIS [19], similar to their application described in [20].
- In the case of GPS Block IIF satellites, pronounced inter-frequency clock biases exist between G5 and G1/G2, with periods of several hours and amplitudes of up to many centimeters. They can be determined from triple-frequency GPS observations from a set of globally distributed GNSS reference stations, as described in [21]. For each of our observation days, we selected 10 globally distributed sites of the IGS (CIBG, DAV1, DGAR, KOUR, MAL2, MKEA, NKLG, NYA2, PNGM, and WTZS) and computed G1/G5—G1/G2 clock corrections, with a temporal resolution of 5 min. We applied them to the CODE GPS Block IIF clock corrections prior to the G1/G5 PPP processing.

The first correction mainly affects the contribution of the code observations to a PPP solution. For the carrier phases, any constant biases are absorbed by the real number ambiguities. Thus, in our applications with at least 30 min of static observations, the first correction has only little effect. The second correction is of importance for GPS Block IIF satellites only, which provide less than half of all dual-frequency GPS/Galileo signals used in our PPP positioning.

**Figure 12:**
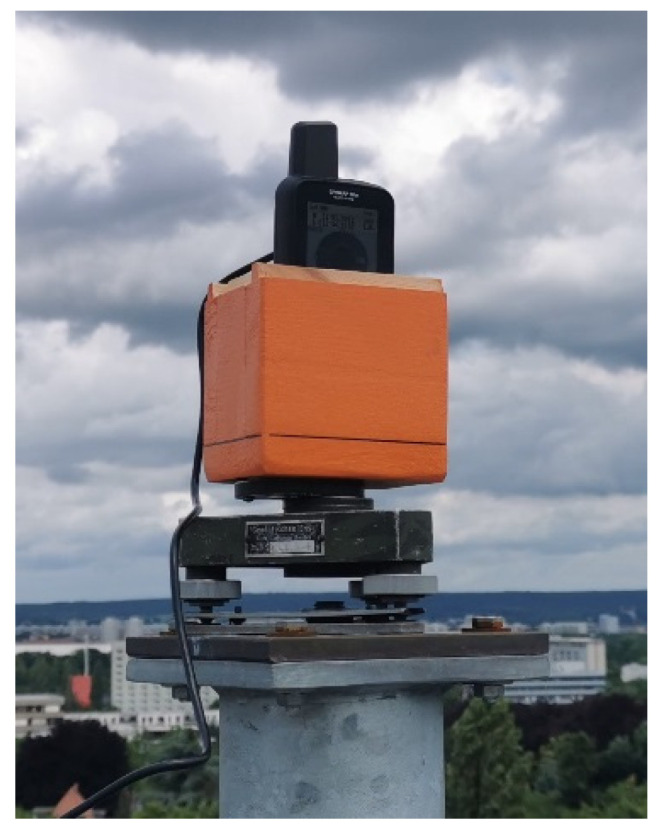
GPSMAP 66sr mounted on tribrach and holder for long-term observations in roof-top environment.

In a first PPP trial, we conducted long-term observations in several sessions of altogether 100 h at a roof-top site without any signal obstructions and with little multipath disturbances. The GPSMAP 66sr was mounted on the tribrach and holder to guarantee horizontal and vertical eccentricities in the order of a few millimeters (Figure 12). An external power supply was used to enable a session length of longer than 24 h.

**Figure 13:**
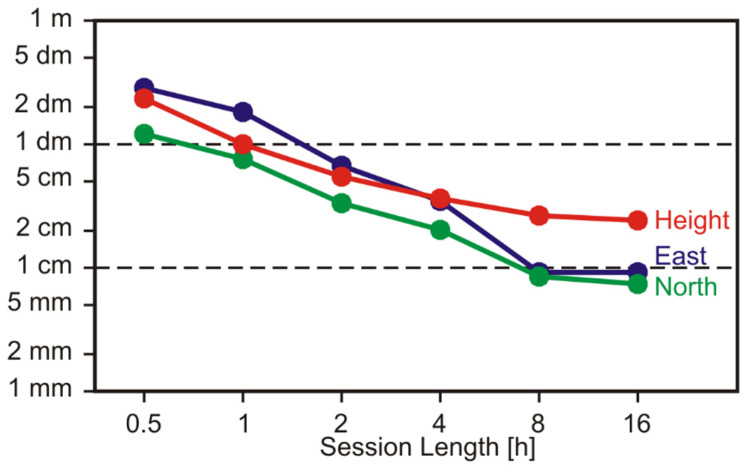
PPP coordinate errors (RMS values) as a function of session length for the static, roof-top observations.

The datasets were divided into sessions of various lengths from 30 min (*n* = 200) to 16 h (*n* = 5). The PPP results were compared with ITRF 2014 station coordinates from earlier GNSS determination with geodetic-grade equipment. RMS values of the deviation in north/east/height are presented in Figure 13. As usual with PPP solutions without ambiguity fixing, so-called float solutions, the north component is determined best, cf. [22]. East and height components show lower accuracies in the sessions up to 4 h. In the longer sessions, the RMS values of the east component become closer to those of the north component. The height component remains worse. With half an hour of observations, the accuracies are on a few 10 cm levels (RMS). However, after several hours, the horizontal components reach 1 cm levels. The RMS values of the height component remain on the few cm levels.

We conclude that, with this kind of Garmin receiver, ITRF coordinates can be determined in PPP mode on the accuracy (RMS) level of 10 cm after about 1 h, 5 cm after a few hours, and a few centimeters after many hours of static observations at a favorable site.

**Figure 14:**
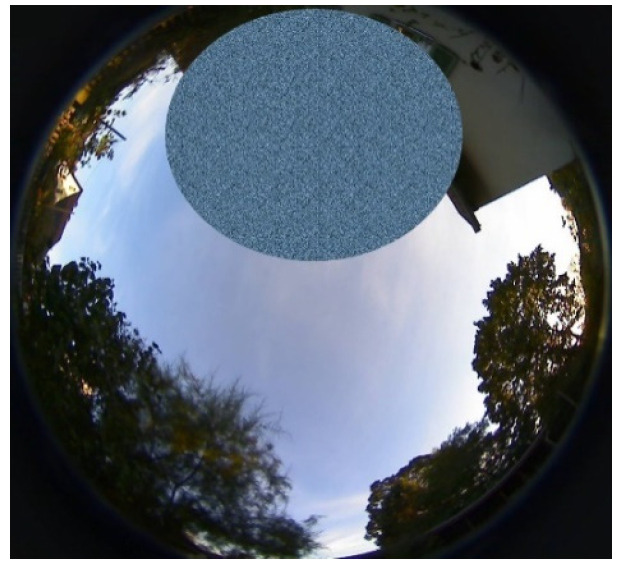
Limited sky visibility at the adverse observation site due to signal obstructions and the satellite visibility gap around the north celestial pole.

A second PPP trial was performed under adverse conditions caused by limited sky visibility. Due to vegetation and a building close by, large portions of the sky were obstructed (Figure 14). Additionally, the satellite visibility gap around the north celestial pole due to satellite orbit inclination angles of nominal 55° (GPS) and 56° (Galileo) contributes to the reduced number of available GNSS signals. The average number of dual-frequency observations above an elevation mask angle of 10 degrees amounted to 6.5. This is considerably lower than the average number of eight, which was demonstrated for the unobstructed site (cf. Figure 3). In addition, some signals penetrated the foliage, which causes signal disturbances, which, in turn, cause, e.g., an increased number of cycle slips.

**Figure 15:**
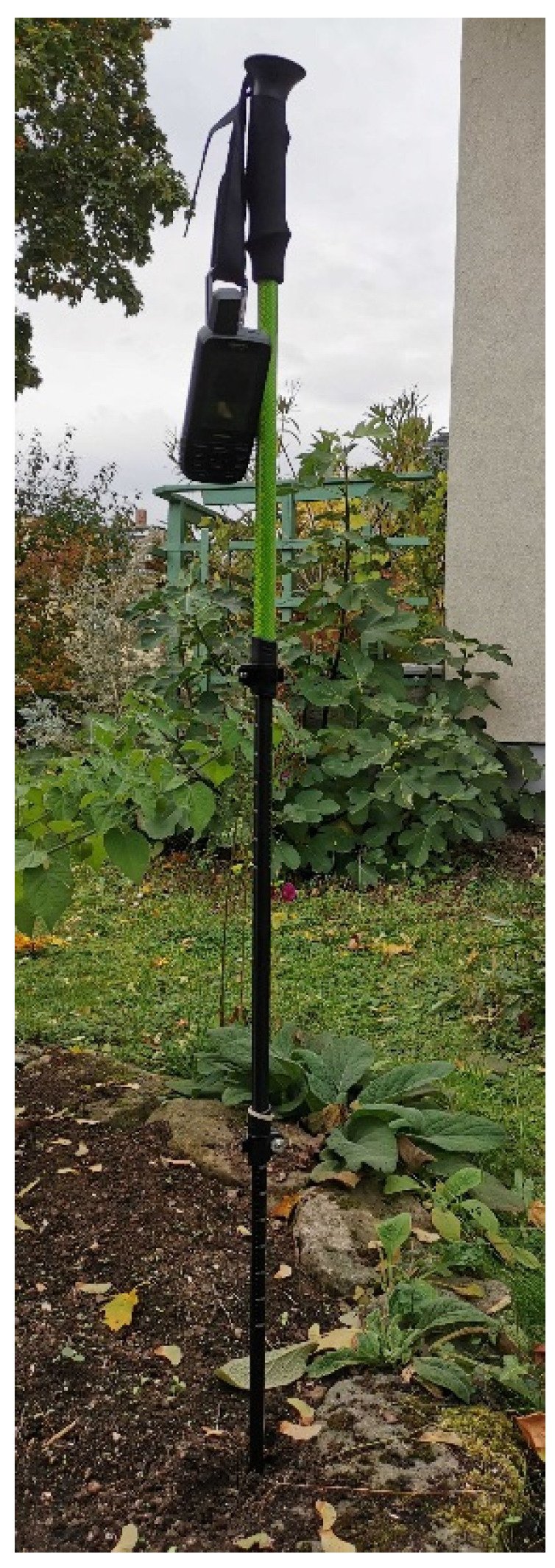
Receiver setup at the adverse site with the help of a trekking pole.

The antenna setup was very much simplified to just fixing the device to the hand strap of a trekking pole (Figure 15). We expect that the eccentricities of the ARP are stable on the level of several centimeters, with larger uncertainties for the horizontal components, as compared with the vertical component. Such an antenna setup should be good enough for positioning accuracies on the level of 10 cm.

We collected data in several sessions of up to 16 h, more than 60 h altogether. The power supply was provided by the internal battery. The PPP results were compared with centimeter-accurate ITRF 2014 coordinates determined by short baselines to a local reference station. The datasets were divided into sessions of various lengths from 30 min (*n* = 126) to 8 h (*n* = 8). Some sessions had to be discarded since the number of tracked GPS/Galileo satellites with dual-frequency observations amounted on average to less than five. The percentages of discarded sessions reached up to 20% for the shorter session lengths. No session had to be discarded from the set of the 8 h sessions.

**Figure 16:**
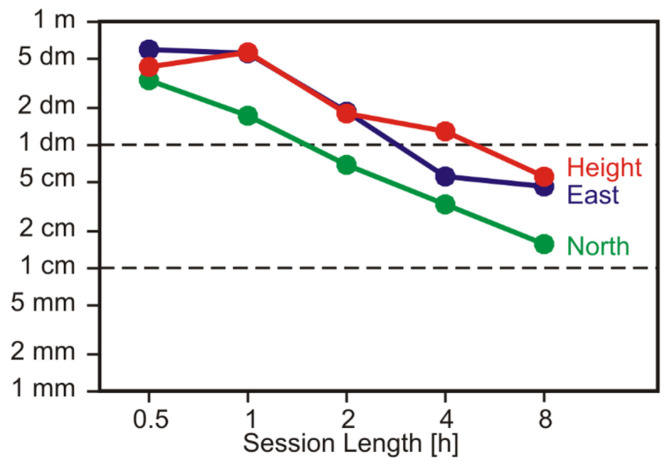
PPP coordinate errors (RMS values) as a function of session length for the static observations under adverse environmental conditions.

The RMS values of coordinate deviations of the remaining sessions are presented in Figure 16. The overall position accuracy is now considerably lower than that in our first trial at the roof-top site. Again, the north component is determined more accurately than the east and height components. After just 30 min, the achieved accuracy is on the level of several 10 cm. A horizontal accuracy on the level of 10 cm (RMS) is obtained after 4 h, with even longer observation durations the results keep improving.

We conclude that, with such a simple setup and with many hours of observations (virtually overnight), the GPSMAP 66sr is capable of providing ITRF coordinates on the level of 10 cm even at an unfavorable GNSS site.

### 4.2. Relative Carrier-Phase Positioning with Respect to VRS

Shorter observation times for centimeter-accurate positioning can be expected in baseline mode. This requires a local reference station or, in our case, a regional network of consciously operating reference stations. The network we used is operated by the Saxonian State Survey Department (Staatsbetrieb Geobasisinformation und Vermessung Sachsen (GeoSN)), with interstation distances of roughly 50 km. This department offers real-time correction data and also runs a postprocessing service SAPOS-GPPS PrO for RINEX files from rover sites [23]. Nowadays, these services are free of charge.

The postprocessing service computes virtual reference station (VRS) observations for the approximate user position (usually a few m off the final user position) and afterward runs a baseline processing between VRS and rover observations. More on this technique can be found, e.g., in [24]. The resulting coordinates of this service refer to the geodetic reference system ETRS89.

SAPOS-GPPS-PrO maintains a database of antenna phase-center corrections, which, of course, did not include the GPSMAP 66sr antenna. The service operators agreed to help by adding our calibration results (Section 3.3) to this database. Afterward, we could send the slightly modified Garmin RINEX files to the processing service and obtained positioning results. The modification of the RINEX files consisted of two changes to the RINEX header records: we added the antenna name and the vertical antenna height between marker and ARP.

**Figure 17:**
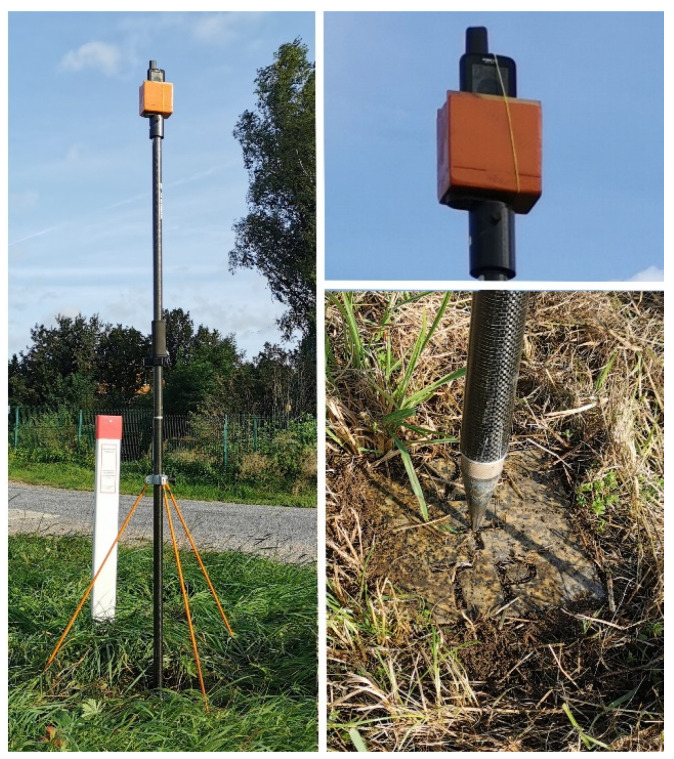
Receiver setup at geodetic control points on top of a 2 m pole.

This relative carrier-phase positioning was tested on five geodetic control points established by GeoSN. We selected control points with minimal or little obstructions and, thus, suitable for precise GNSS positioning. The primary station marks were buried 80 cm down. However, the stations come with a stone monument as surface marks for ordinary use. We mounted the Garmin receiver in its holder on top of a 2 m pole (Figure 17). This setup allows the determination of vertical eccentricities to better a few mm and of the horizontal eccentricities better 1 to 2 cm.

At each station, we collected observations for more than 1 h. The RINEX files were cut into files of 15 min of observations, four files per station. Each file was sent to the SAPOS-GPPS PrO processing service, and in all cases, we received in return ambiguity-fixed solutions with quality indicator “high”. We compared these results with the ETRS89 station coordinates from the GeoSN database. These coordinates possess the accuracy levels: horizontal <2cm and vertical +−3 cm.

**Figure 18:**
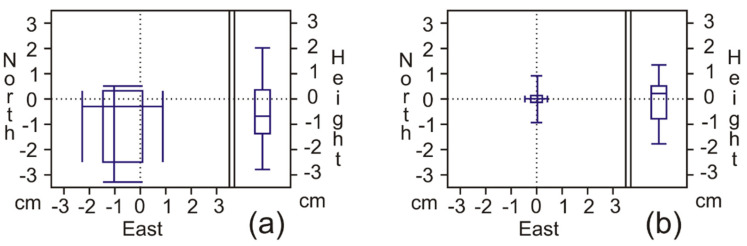
Distribution of coordinate errors of 15 min sessions (*n* = 20) as 2D (north/east) and 1D (height) box-and-whisker plots depicting percentile values for 0 (minimum), 25, 50 (median), 75, and 100 (maximum): (**a**) absolute errors with respect to station coordinates of the state survey department, (**b**) repeatability of the 4 determinations per station.

In a first comparison (Figure 18a), we determined the coordinate errors with respect to the provided station coordinates. All deviations stayed within +−3 cm. The main causes of these deviations are the limited accuracy of the given ETRS89 station coordinates, eccentricity errors of our setup, especially in the horizontal components, and GNSS positioning errors. With these trial data, we were not able to separate these three contributions from another, but nevertheless, we can conclude that centimeter-accurate positioning can be achieved.

In order to get more insight into the GNSS positioning errors, we also compared the 15 min session results with the mean ETRS89 positions determined with 1 h of observations (Figure 18b). Since the eccentricities remained unchanged, the 15 min session results are only independent with respect to GNSS. The horizontal deviations now clearly demonstrate sub cm accuracy. The vertical deviations are slightly larger.

We conclude that, in relative carrier-phase positioning mode with session lengths of many minutes, the GPSMAP 66sr is capable of reliably providing coordinate results on the centimeter level.

## 5. Conclusions and Outlook

The Garmin GPSMAP 66sr (v3.30) offers dual-frequency GNSS observations for cm-level postprocessing applications. Due to its quadrifilar helix antenna, the observation quality exceeds the quality of other consumer devices with dual-frequency capability, e.g., smartphones. Centimeter-level positioning results can be achieved in long observation sessions (several hours), with precise point positioning (PPP), or in short observation sessions (many minutes) in baseline mode.

The main limitation of the device with respect to precise GNSS positioning is caused by the incompleteness of the tracked satellite signals. In particular, a higher priority of Galileo in the satellite selection process would considerably improve precise positioning results. If this problem is solved and with an increasing number of active Galileo satellites and more GPS satellites capable of transmitting G5 signals in the near future, the positioning performance will even exceed the level demonstrated in our trials.

## Data Availability

A RINEX sample dataset of Garmin GPSMAP 66sr is available from the corresponding author on request. All products of the International GNSS Service (IGS) or of one of the IGS analysis centers were obtained from CDDIS. The observation data of the virtual reference stations (VRS) were made available by Staatsbetrieb Geobasisinformation und Vermessung Sachsen (GeoSN).

## Supplementary Materials

The following are available online at https://www.mdpi.com/article/10.3390/s22051964/s1, File S1: Antenna calibration results in ANTEX format.
